# Will SARS-CoV-2 Become Just Another Seasonal Coronavirus?

**DOI:** 10.3390/v13050854

**Published:** 2021-05-07

**Authors:** Alexander B. Beams, Rebecca Bateman, Frederick R. Adler

**Affiliations:** 1 Department of Mathematics, University of Utah, Salt Lake City, UT 84112, USA; 2 Division of Epidemiology, University of Utah, Salt Lake City, UT 84108, USA; adler@math.utah.edu; 3 University of Utah, Salt Lake City, UT 84112, USA; rbateman@asuu.utah.edu; 4 School of Biological Sciences, University of Utah, Salt Lake City, UT 84112, USA

**Keywords:** SARS-CoV-2, mathematical model, ordinary differential equations, SIR model

## Abstract

The future prevalence and virulence of SARS-CoV-2 is uncertain. Some emerging pathogens become avirulent as populations approach herd immunity. Although not all viruses follow this path, the fact that the seasonal coronaviruses are benign gives some hope. We develop a general mathematical model to predict when the interplay among three factors, correlation of severity in consecutive infections, population heterogeneity in susceptibility due to age, and reduced severity due to partial immunity, will promote avirulence as SARS-CoV-2 becomes endemic. Each of these components has the potential to limit severe, high-shedding cases over time under the right circumstances, but in combination they can rapidly reduce the frequency of more severe and infectious manifestation of disease over a wide range of conditions. As more reinfections are captured in data over the next several years, these models will help to test if COVID-19 severity is beginning to attenuate in the ways our model predicts, and to predict the disease.

## 1. Introduction

The future of SARS-CoV-2 is uncertain, and it is tempting to look for parallels in the dramatic animal-to-human crossovers of SARS-like coronaviruses that have occurred in the last twenty years [1,2]. However, neither SARS or MERS had the pandemic potential of SARS-CoV-2, which will likely persist alongside the seasonal coronaviruses [3]. A better clue to the future severity of COVID-19 infection can be found by examining the manner in which pathogens tend toward avirulence over time. For example, genetic analysis suggests that a pandemic in the 1890’s historically attributed to influenza may in fact have been caused by the seasonal coronavirus OC43 when it first began circulating in humans, and the fact that OC43 is just another seasonal coronavirus today suggests SARS-CoV-2 could follow a similar path [4]. Additionally, SARS-CoV-2 resembles its benign cold-causing relatives in some important ways. Coronavirus NL63 uses the same ACE2 receptor to enter highly differentiated epithelial cells [5,6]. Yet NL63 too is just another seasonal coronavirus [2].

The severity of COVID-19 is changing as genetic variants of SARS-CoV-2 undergo natural selection, but genetic evolution is not the only means by which disease severity can attenuate over time. Suggestive evidence for non-genetic change is given by the experiences of the British Antarctic Survey who, after returning to society after long periods of isolation in the polar environment, experienced fairly severe disease from rhinovirus infection [7], the most common cause of the common cold [8]. Because reliable immune protection against severe disease seems to require frequent antigen exposure, the immune response to forgotten or novel viruses may be ineffective and the symptoms due to immunopathology [9]. Thus, on a short timescale before natural selection has much time to act, the severity of disease may decrease as populations collectively develop immunity.

In this paper, we use mathematical models to characterize the conditions under which the immune system can promote avirulence on this short timescale, rendering SARS-CoV-2 into Just Another Seasonal Coronavirus (JASC). Our models include three factors that might push SARS-CoV-2 towards becoming JASC. First, mild or asymptomatic cases tend to shed less virus, creating smaller infectious doses and subsequent mild infections. Second, children rarely experience more severe forms of COVID-19, and thus may shed less virus and develop protective immunity. Third, the duration and strength of immunity to SARS-CoV-2 could generate mild cases as the population develops an umbrella of partial immunity much like that to the other common cold viruses.

### 1.1. Small SARS-CoV-2 Doses Are Associated with Mild COVID-19 Disease

Severe cases of COVID-19 might result from high doses of virus particles overwhelming the immune system [10]. Specifically, high doses might outpace or overwhelm the initial T cell response, leading to immunosuppression and severe illness before the seroconversion process begins, as opposed to rapid clearance and milder illness [2]. Regardless of mechanism, there is a laboratory-confirmed dose response for infection severity in hamsters, both for SARS and SARS-CoV-2 [11,12]. Mild forms of illness are associated with short-duration exposures in less crowded, more open, and better ventilated spaces [13]. In China, severe cases correlated with being near the epicenter of the outbreak as opposed to lower-transmission settings [14]. Higher SARS-CoV-2 viral loads correlate with severe COVID-19 and death in adults [15]. In addition to higher viral loads, severe cases shed virus particles for longer periods of time [16]. Asymptomatic individuals transmit SARS-CoV-2 but may be less infectious [17]. Mild or asymptomatic rhinovirus infection is associated with lower viral loads as well [18]. A mathematical modeling study which accounted for a dose response in disease severity attributed the second, deadlier wave of the 1918 influenza pandemic to crowding of infectious individuals as opposed to viral evolution [19].

### 1.2. Children Develop Mild COVID-19 Infections

Children experience milder forms of SARS-CoV-2 infection [20,21,22], as in the SARS outbreak of 2004 [23]. Coronaviruses in general seem to thrive in disrupted immune systems, and younger people may exhibit a more robust response than immunosenescent individuals [24,25,26] thanks to their more robust immune systems with more T cells and NK cells [27]. Children also tend to have fewer comorbidities than adults [20], and may be less susceptible to SARS-CoV-2 infection in the first place [28]. Despite the fact that children do not seem to drive much transmission [29], infected children without symptoms have viral burdens comparable to or greater than hospitalized adults with severe COVID-19 [30]. In contrast, the most common seasonal coronaviruses (229E and OC43) appear to mostly infect children [31]. When stratified by age, people between the ages of 15–19 have the highest positivity rate for seasonal coronaviruses and infants aged 7–11 months have the lowest [32]. Children drive transmission of some pathogens (like rhinovirus) due to mixing patterns and behavior [33]. Infection by chicken pox, measles, polio, and Epstein-Barr tend to produce less severe symptoms in children [34,35,36,37] although RSV provides a notable exception [38].

### 1.3. Partial Immunity Results in Mild Disease

Immune protection is antigen-dependent, with more recent viral exposure translating to milder illness [9]. The vast majority of seasonal coronavirus infections are asymptomatic and up to 45% of all SARS-CoV-2 infections could be asymptomatic [39,40]. Reinfection by seasonal coronaviruses is common [41]. Indeed, most people carry antibodies for all of the seasonal coronaviruses, which likely confer some degree of immunity to that specific strain in addition to some cross-immunity between closely related strains [41]. The coronaviruses that produce SARS-like symptoms tend to rely on disrupting the interferon response [42]. Exposure to prior antigens might stimulate the interferon response and other components of the innate pathway early in infection, keeping viremia under control and resulting in mild infection [43].

### 1.4. Non-Pharmaceutical Interventions (NPI) Promote Mild Disease

At the beginning of the pandemic, travel restrictions to and from Wuhan were put in place to contain the virus. Although that was not sufficient to halt spread, mathematical models fit to data showed that the reproduction number decreased by 50% [44]. Since then, a combination of non-pharmaceutical interventions (NPI) have been employed with varying degrees of sucess across the world to keep the infection rate low, allowing the population to approach herd immunity at a controlled pace as vaccines are employed (“flattening the curve”) [45]. Modeling work suggests that social distancing, mask use, and school closures in combination (but not in isolation) can limit the spread of the virus until the population is vaccinated [46]. Masks, cleaning, ventilation, and social distancing all serve to reduce infecting doses of virus particles [13,47]. Epidemiological data suggest that masks are the most effective components of the NPI bundle to limit infections [48]. Laboratory experiments have confirmed that surgical mask partitions separating infected and uninfected hamsters confer protection against severe illness [12]. Similarly, social distancing limits transmission by creating more diffuse infectious viral doses, with the consequence that individuals who do get infected in more open spaces tend to develop milder infections [49]. NPI’s have modulated age-dependent transmission pathways to control viral spread as well. Early in the pandemic, school closures were thought to be a safeguard against an accelerating rise in cases [50]. Whether school closures have significantly reduced deaths from COVID-19 is disputed [51].

### 1.5. Vaccines

Natural and vaccine-induced immune memory fades over time, rendering individuals more susceptible to infection and severe illness. Vaccines that do not confer sterilizing immunity may still boost individuals into a partially immune class which has reduced susceptibility for infection and increased likelihood of mild illness [52]. Waning immune memory could permit viruses to transmit more effectively, as is the case for Varicella [53]. Additionally, vaccines seem to stimulate general immune pathways which prime the immune system to respond to other pathogens [43]. Collectively, this suggests that even if sterilizing immunity wanes over a time, a history of vaccination or viral exposure may predispose individuals to develop milder secondary infections by SARS-CoV-2 because the immune system will mount a more robust response.

We first formulate and analyze a simple model that incorporates heterogeneity in the infection phenotype (high- and low-shedding infections) and immunity that wanes into partial immunity, and then extend it to a full model with age structure in the population and vaccination. To quantify whether SARS-COV-2 becomes JASC, we assess the number of severe infections during both the initial epidemic and over the long run. We assess how our three hypothesized factors (dose effect, less severe infections in children, and the effect of partial immunity) individually and collectively reduce the number of severe cases, and whether they act synergistically.

## 2. Methods

In order to understand how a dose response, partial immunity, and children can push SARS-CoV-2 towards JASC, we first need to understand their effects independently. To this end, we begin with a model of the spread of a virus which can cause two types of infection, followed by a simple age-structured model which only tracks a single type of infection. We obtain some analytical results in these cases. Then, we combine these models into an age-structured model that tracks the spread of two types of infection and examine it numerically.

### 2.1. Model with Two Infection Types

In this model, susceptible individuals (*S*) can acquire two types of infection: “high-shedding” (*H*) and “low-shedding” (*L*). In either case, individuals remain infected for an average of 1/γ days before gaining sterilizing immunity (*R*). Sterilizing immunity wanes with a rate of ρ/day, rendering individuals partially immune (*P*). Partial immunity wanes at rate $\tilde{\rho }$/day. The system of equations is given by

$$\begin{array}{cc} \frac{dS}{dt} & =-\alpha \left(\theta H+L\right)S+\tilde{\rho }P,\\ \frac{dP}{dt} & =-\alpha \tilde{\nu }\left(\theta H+L\right)P+\rho R-\tilde{\rho }P,\\ \frac{dH}{dt} & =\alpha \left(1-Q_{h}\right)S\theta H+\alpha \left(1-Q_{l}\right)SL-\gamma H+\alpha \left(1-{\tilde{Q}}_{h}\right)\tilde{\nu }P\theta H+\alpha \left(1-{\tilde{Q}}_{l}\right)\tilde{\nu }PL,\\ \frac{dL}{dt} & =\alpha Q_{h}S\theta H+\alpha Q_{l}SL-\gamma L+\alpha {\tilde{Q}}_{h}\tilde{\nu }P\theta H+\alpha {\tilde{Q}}_{l}\tilde{\nu }PL,\\ \frac{dR}{dt} & =\gamma (L+H)-\rho R,\\ N & =S+P+H+L+R. \end{array}$$

For the preliminary analysis, we make the simplifying assumptions that both infection types are equally infectious (θ=1) and that partially immune individuals are infected at the same per capita rate α as susceptible individuals ($\tilde{\nu }=1$). The parameters $Q_{l}$ and $Q_{h}$ represent the probability that a susceptible individual develops the low-shedding form of infection when exposed to low- or high-shedding infections respectively. ${\tilde{Q}}_{l}$ and ${\tilde{Q}}_{h}$ represent the same probabilities for partially immune individuals. When partial immunity protects against the high-shedding form of infection, ${\tilde{Q}}_{l}>Q_{l}$ and ${\tilde{Q}}_{h}>Q_{h}$. Because we expect that low-shedding infections tend to generate low-shedding infections, we investigate cases with $Q_{l}\geq Q_{h}$ and ${\tilde{Q}}_{l}\geq {\tilde{Q}}_{h}$.

When $\gamma >>\rho ,\tilde{\rho }$ the steady-state solution for this system following an outbreak is approximately

$$\begin{array}{cc} S^{*} & =N\frac{1}{R_{0}}\frac{\tilde{\rho }}{\tilde{\rho }+\rho \left(R_{0}-1\right)},\\ P^{*} & =N\frac{1}{R_{0}}\frac{\rho (R_{0}-1)}{\tilde{\rho }+\rho \left(R_{0}-1\right)},\\ L^{*} & =0,\\ H^{*} & =0,\\ R^{*} & =N\left(1-\frac{1}{R_{0}}\right). \end{array}$$

where $R_{0}=\alpha N/\gamma$. The $P^{*}$ equation shows that greater transmissibility produces more partial immunity in the population. Because we assume that *L* and *H* are equally infectious and *S* and *P* are equally susceptible, the total number of infections *L* + *H* matches the equilibrium of a standard SIR model [54],

$$\begin{array}{cc} {\left(H+L\right)}^{*} & =N\frac{\rho }{\gamma +\rho }\left(1-\frac{1}{R_{0}}\right). \end{array}$$

A longer duration of immunity, created by a smaller loss of immunity ρ, implies fewer infection at steady state, matching the approximation above. Because this expression is independent of the *Q* parameters, these parameters affect only the relative number of infections of the two types at steady state. If we split the *R* compartment into two different states, $R_{h}$ and $R_{l}$, to track the numbers of immune individuals who pass through the *H* or *L* compartments respectively, it is not hard to verify that $R_{l}^{*}=\left(\gamma /\rho \right)L^{*}$, and $R_{h}^{*}=\left(\gamma /\rho \right)H^{*}$.

If exposure to high-shedding infections always produces high-shedding infections ($Q_{h}={\tilde{Q}}_{h}=0$) and low-shedding infections produce at least some high-shedding infections, then low-shedding infections will eventually disappear ($L^{*}=0$, see full steady state solution in Appendix A). Conversely, if exposure to low-shedding infections always produces low-shedding infections ($Q_{l}={\tilde{Q}}_{l}=1$) and high-shedding infections produce at least some low-shedding infections, then high-shedding infections will disappear ($H^{*}=0$). If each type of infection seeds only itself, the steady state equations are degenerate with values determined by the initial conditions.

**Figure 1:**
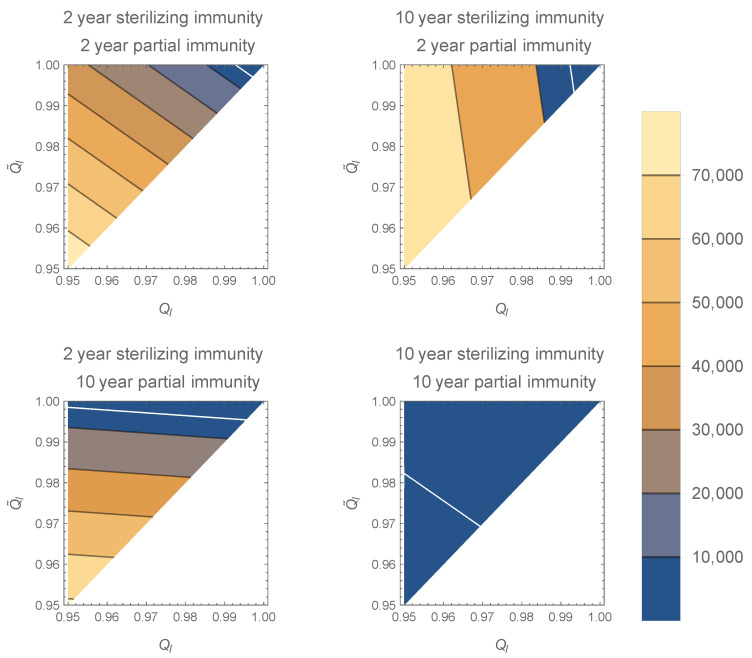
Contour plots of $H^{*}$ in the model with two infection types for various $Q_{l}$ and ${\tilde{Q}}_{l}$ along the axes. The white line corresponds to $H^{*}=5000$. As $Q_{l}$ and ${\tilde{Q}}_{l}$ approach 1, $H^{*}$ decreases to 0 (top right corner of plots). If sterilizing immunity is long compared to partial immunity, $H^{*}$ is more sensitive to increases in $Q_{l}$, especially near 0. If partial immunity is long compared to sterilizing immunity, $H^{*}$ is more sensitive to ${\tilde{Q}}_{l}$, especially near 0. Increasing the duration of sterilizing immunity decreases $H^{*}$. All plots generated with $R_{0}=2.5$, $N=10^{8}$, $Q_{h}=0,{\tilde{Q}}_{l}=0.8$, and γ=1/10 per day.

The contour plot in Figure 1 shows that the equilibrium number of high-shedding cases, $H^{*}$, is lowest when both full and partial immunity last 10 years. As shown above, $H^{*}$ decreases to 0 as $Q_{l}$ and ${\tilde{Q}}_{l}$ approach 1 for all values of ρ and $\tilde{\rho }$. The equilibrium is most sensitive to the value of $Q_{h}$ when the duration of full immunity is long, and to the value of ${\tilde{Q}}_{l}$ when the duration of partial immunity is long.

The model’s transient dynamics can differ dramatically from the steady state. In the limiting case where exposure to high-shedding infection always results in high-shedding infection for susceptible individuals and all other exposures lead to low-shedding infection ($Q_{h}=0$, ${\tilde{Q}}_{h}=1$, $Q_{l}=1$, ${\tilde{Q}}_{l}=1$), $H^{*}=0$ will be zero and all of the endemic infection will low-shedding. However, the initial outbreak looks very different. From an initial epidemic seeded by a single high-shedding case (S(0)=N−1, H(0)=1,L(0)=0,P(0)=0,R(0)=0), and neglecting the small rates of immune loss because ρ<<γ, and $\tilde{\rho }<<\gamma$, the system is approximately given by

$$\begin{array}{cc} \frac{dS}{dt} & =-\alpha HS,\\ \frac{dP}{dt} & =0,\\ \frac{dH}{dt} & =\alpha SH-\gamma H,\\ \frac{dL}{dt} & =0,\\ \frac{dR}{dt} & =\gamma \;H\\ N & =S+H+R. \end{array}$$

The initial outbreak matches a standard SIR model consisting only of high-shedding infections, *H*. The final size relationship between $R_{0}$ and the fraction of the population experiencing infection, *f*, is given by

$$\begin{array}{cc} R_{0} & =\frac{-\ln(1-f)}{f}. \end{array}$$

Values of $R_{0}$ near 2.5 give f≈0.9.

On a longer timescale, about 1/ρ weeks after the initial outbreak, a subsequent outbreak can begin because a substantial portion of the immune fraction, *f*, have lost sterilizing immunity. However, these individuals enter into the partially immune compartment *P*, not *S*. When *f* is close to 1, as it will be with $R_{0}\approx 2.5$, the outbreak will follow the same dynamics but consist almost entirely of low-shedding cases from the partially immune class.

$$\begin{array}{cc} \frac{dS}{dt} & =0,\\ \frac{dP}{dt} & =-\alpha LP,\\ \frac{dH}{dt} & =0,\\ \frac{dL}{dt} & =-\gamma L+\alpha PL,\\ \frac{dR}{dt} & =\gamma L,\\ N & =P+L+R. \end{array}$$

**Figure 2:**
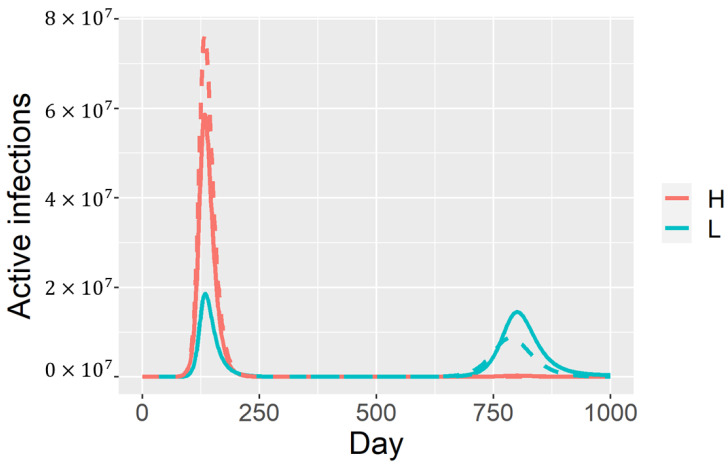
Partial immunity and a dose response can promote avirulence.The initial outbreak consists mainly of high-shedding cases, but these are replaced by low-shedding cases in subsequent outbreaks. The dashed curve corresponds to the approximate dynamics described in the text. Solid curves are model solutions under the parametrization ϵ=0.01, where $Q_{ah}=\epsilon$ and $Q_{al}=Q_{kl}=Q_{kh}=\tilde{Q_{a}h}=\tilde{Q_{a}l}=\tilde{Q_{k}h}=\tilde{Q_{k}l}=1-\epsilon$.

In this idealized case, no high-shedding *H* infections will accrue during the second outbreak. The compartment of partially immune individuals *P* serves as a large reservoir of *L* infections. In the more realistic case with $R_{0}=2.5$, the actual waves overlap, and we expect severe cases to drop by 90% in this wave, and by a similar fraction in subsequent waves (Figure 2).

Modifications of the *Q* parameters away from 0 and 1 render this approximation less accurate, but the behavior of the model is qualitatively similar. As long as the buildup of partial immunity is sufficiently rapid, the *H* infections will be largely replaced by *L* infections even though the initial wave has few, if any, *L* infections.

**Figure A1:**
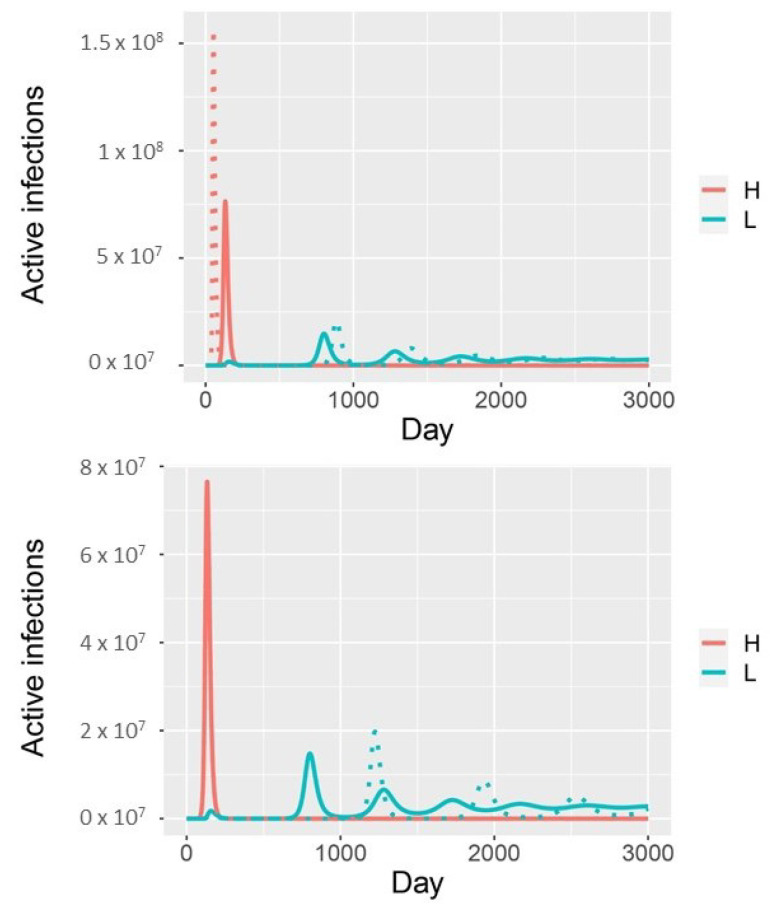
Accounting for infectiousness θ and susceptibility $\tilde{\nu }$ in the model with two infection phenotypes If high-shedding individuals are twice as infectious, the initial outbreak is larger, consists of fewer low-shedding cases, and the subsequent outbreak of low-shedding cases occurs later (dotted curve: θ=2, solid curve: θ=1, (**top panel**)). If partially immune individuals are half as susceptible, it takes longer to reach the endemic steady state because subsequent outbreaks occur later (dotted curves: $\tilde{\nu }=1/2$, solid curves: $\tilde{\nu }=1$, (**bottom panel**); all other parameters as in Figure 2).

The long-term behavior of the model does not substantially change if θ or $\tilde{\nu }$ deviate from 1. If high-shedding cases are more infectious (θ>1) the initial outbreak of high-shedding cases will be larger, and occur slightly sooner. The subsequent outbreak of low-shedding cases will occur later (Figure A1). If partially immune individuals are less susceptible ($\tilde{\nu }<1$) the initial outbreak is unaffected but subsequent outbreaks occur later, and the system takes longer to reach the endemic steady state.

### 2.2. Model with Age-Structure Only

In the second model, we revert to a single-phenotype infection but account for variation in susceptibility across the population by splitting the population into two interacting groups, interpreted here as age structure (young and old), although other stratifications according to susceptibility would be equally well described by the same equations. One group is indexed with “*k*” (children) and the other by “*a*” (adults). The equations are

$$\begin{array}{cc} \frac{dS_{a}}{dt} & =-\alpha \left(I_{a}+I_{k}\right)S_{a}+\rho R_{a},\\ \frac{dS_{k}}{dt} & =-\alpha \nu \left(I_{a}+I_{k}\right)S_{k}+\rho R_{k},\\ \frac{dI_{a}}{dt} & =\alpha \left(I_{a}+I_{k}\right)S_{a}-\gamma I_{a},\\ \frac{dI_{k}}{dt} & =\alpha \nu \left(I_{a}+I_{k}\right)S_{k}-\gamma I_{k},\\ \frac{dR_{a}}{dt} & =\gamma I_{a}-\rho R_{a},\\ \frac{dR_{k}}{dt} & =\gamma I_{k}-\rho R_{k},\\ N_{a} & =S_{a}+I_{a}+R_{a},\\ N_{k} & =S_{k}+I_{k}+R_{k}. \end{array}$$

**Figure 3:**
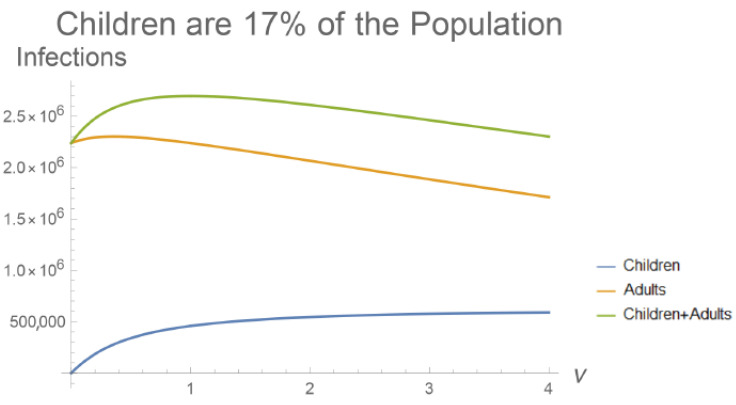
The values of $I_{a}^{*}$ and $I_{k}^{*}$ from the age-structured model plotted against ν, the relative susceptibility of children. Total infections are maximized when children are equally susceptible to adults (ν=1). Infections are most concentrated in adults if children are less susceptible (ν<1). Children reduce infections in adults if they are more susceptible (ν>1). These relationships are more pronounced if children make up a greater share of the population (Figure A2). The population of the United States is $N_{a}+N_{k}$ = 328,239,523, and children are considered under 14 years of age ($N_{k}$ = 56,406,387) [55].

The parameter ν represents the susceptibility of children relative to adults, which can be smaller or larger than 1 to model reduced or enhanced susceptibility. Although the expressions are much more cumbersome, it is possible to solve this system at steady-state, with infection most concentrated in adults for values of ν which are intermediate between 0 and 1 (Figure 3). The total number of infections in the population is maximized when both groups are equally susceptible. If children are much more susceptible than adults (ν→∞), then the number of infections declines until the entire share of infections is concentrated in children. Reducing $R_{0}$ produces more marked declines in $I_{a}^{*}$ than $I_{k}^{*}$ when children make up 17% of the population, matching the fraction of the US population younger than 14 years (not shown).

On a short timescale when immune loss has negligible impact on the dynamics, it is possible to derive a final-size relation describing the initial outbreak for the two subpopulations, obeying the power law relationship

$$\begin{array}{c} \frac{S_{a,\infty }}{N_{a}}={\left(\frac{S_{k,\infty }}{N_{k}}\right)}^{1/\nu }. \end{array}$$

If children are much less susceptible to infection (ν→0), then more of the adult population experiences infection during the outbreak. The reverse holds true if children are more susceptible than adults, in qualitative agreement with the steady-state analysis in Figure 3.

### 2.3. The Full Model

We combine the previous models to describe the spread of the two infection phenotypes (*H* and *L*) across the two interacting subpopulations (adults and children). There are now twelve state variables: susceptible adults $S_{a}$, low- and high-shedding infectious adults $L_{a}$ and $H_{a}$, immune adults stratified according to recovery from low- or high-shedding infection, $R_{al}$ and $R_{ah}$, and partially immune adults $P_{a}$. Children have the same states, except the state variables have *k* in place of *a* in the subscripts. The system of equations for the adult subpopulation is given by

$$\begin{array}{ccc} \frac{dS_{a}}{dt} & = & \tilde{\rho }P_{a}+\rho R_{al}+\rho R_{ah}-\\ & & \;\alpha_{ak}S_{a}\frac{L_{k}}{N_{k}}-\alpha_{aa}S_{a}\frac{L_{a}}{N_{a}}-\alpha_{ak}\theta S_{a}\frac{H_{k}}{N_{k}}-\alpha_{aa}\theta S_{a}\frac{H_{a}}{N_{a}}+\tau S_{k}-\mu S_{a},\\ \frac{dL_{a}}{dt} & = & \alpha_{ak}S_{a}\frac{L_{k}}{N_{k}}Q_{al}+\alpha_{aa}S_{a}\frac{L_{a}}{N_{a}}Q_{al}+\alpha_{ak}\theta S_{a}\frac{H_{k}}{N_{k}}Q_{ah}+\alpha_{aa}\theta S_{a}\frac{H_{a}}{N_{a}}Q_{ah}+\\ & & \;\alpha_{ak}\tilde{\nu_{a}}P_{a}\frac{L_{k}}{N_{k}}\tilde{Q_{al}}+\alpha_{aa}\tilde{\nu_{a}}P_{a}\frac{L_{a}}{N_{a}}\tilde{Q_{al}}+\alpha_{ak}\theta \tilde{\nu_{a}}P_{a}\frac{H_{k}}{N_{k}}\tilde{Q_{ah}}+\alpha_{aa}\theta \tilde{\nu_{a}}P_{a}\frac{H_{a}}{N_{a}}\tilde{Q_{ah}}+\\ & & \;\tau L_{k}-\gamma_{al}L_{a}-\mu L_{a},\\ \frac{dH_{a}}{dt} & = & \alpha_{ak}\nu_{a}S_{a}\frac{L_{k}}{N_{k}}\left(1-Q_{al}\right)+\alpha_{aa}\nu_{a}S_{a}\frac{L_{a}}{N_{a}}\left(1-Q_{al}\right)+\\ & & \;\alpha_{ak}\theta \nu_{a}S_{a}\frac{H_{k}}{N_{k}}\left(1-Q_{ah}\right)+\alpha_{aa}\theta \nu_{a}S_{a}\frac{H_{a}}{N_{a}}\left(1-Q_{ah}\right)+\\ & & \;\alpha_{ak}\tilde{\nu_{a}}P_{a}\frac{L_{k}}{N_{k}}\left(1-\tilde{Q_{al}}\right)+\alpha_{aa}\tilde{\nu_{a}}P_{a}\frac{L_{a}}{N_{a}}\left(1-\tilde{Q_{al}}\right)+\\ & & \;\alpha_{ak}\theta \tilde{\nu_{a}}P_{a}\frac{H_{k}}{N_{k}}\left(1-\tilde{Q_{ah}}\right)+\alpha_{aa}\theta \tilde{\nu_{a}}P_{a}\frac{H_{a}}{N_{a}}\left(1-\tilde{Q_{ah}}\right)+\\ & & \;\tau H_{k}-\gamma H_{a}-\mu H_{a},\\ \frac{dR_{al}}{dt} & = & \gamma L_{a}+\tau R_{kl}-\rho R_{al}-\mu R_{al},\\ \frac{dR_{ah}}{dt} & = & \gamma H_{a}+\tau R_{kh}-\rho R_{ah}-\mu R_{ah},\\ \frac{dP_{a}}{dt} & = & \rho_{al}R_{al}+\rho_{ah}R_{ah}-\\ & & \;\;\alpha_{ak}\tilde{\nu_{a}}P_{a}\frac{L_{k}}{N_{k}}+\alpha_{aa}\tilde{\nu_{a}}P_{a}\frac{L_{a}}{N_{a}}+\alpha_{ak}\theta \tilde{\nu_{a}}P_{a}\frac{H_{k}}{N_{k}}+\alpha_{aa}\theta \tilde{\nu_{a}}P_{a}\frac{H_{a}}{N_{a}}+\tau P_{k}-\tilde{\rho }P_{a}-\mu P_{a}, \end{array}$$

and the system of equations describing the spread of infections within the subpopulation of children is given by

$$\begin{array}{ccc} \frac{dS_{k}}{dt} & = & \tilde{\rho }P_{k}+\rho R_{kl}+\rho R_{kh}-\\ & & \;\alpha_{kk}\nu_{k}S_{k}\frac{L_{k}}{N_{k}}-\alpha_{ak}\nu_{k}S_{k}\frac{L_{a}}{N_{a}}-\alpha_{kk}\theta \nu_{k}S_{k}\frac{H_{k}}{N_{k}}-\alpha_{ak}\theta \nu_{k}S_{k}\frac{H_{a}}{N_{a}}-\tau S_{k}-\mu S_{k},\\ \frac{dL_{k}}{dt} & = & \alpha_{kk}\nu_{k}S_{k}\frac{L_{k}}{N_{k}}Q_{kl}+\alpha_{ak}\nu_{k}S_{k}\frac{L_{a}}{N_{a}}Q_{kl}+\alpha_{kk}\theta \nu_{k}S_{k}\frac{H_{k}}{N_{k}}Q_{kh}+\alpha_{ak}\theta \nu_{k}S_{k}\frac{H_{a}}{N_{a}}Q_{kh}+\\ & & \;\alpha_{kk}\tilde{\nu_{k}}P_{k}\frac{L_{k}}{N_{k}}\tilde{Q_{kl}}+\alpha_{ak}\tilde{\nu_{k}}P_{k}\frac{L_{a}}{N_{a}}\tilde{Q_{kl}}+\alpha_{kk}\theta \tilde{\nu_{k}}P_{k}\frac{H_{k}}{N_{k}}\tilde{Q_{kh}}+\alpha_{ak}\theta \tilde{\nu_{k}}P_{k}\frac{H_{a}}{N_{a}}\tilde{Q_{kh}}-\\ & & \;\tau L_{k}-\gamma L_{k}-\mu L_{k},\\ \frac{dH_{k}}{dt} & = & \alpha_{kk}\nu_{k}S_{k}\frac{L_{k}}{N_{k}}\left(1-Q_{kl}\right)+\alpha_{ak}\nu_{k}S_{k}\frac{L_{a}}{N_{a}}\left(1-Q_{kl}\right)+\\ & & \;\alpha_{kk}\theta \nu_{k}S_{k}\frac{H_{k}}{N_{k}}\left(1-Q_{kh}\right)+\alpha_{ak}\theta \nu_{k}S_{k}\frac{H_{a}}{N_{a}}\left(1-Q_{kh}\right)+\\ & & \;\alpha_{kk}\tilde{\nu_{k}}P_{k}\frac{L_{k}}{N_{k}}\left(1-\tilde{Q_{kl}}\right)+\alpha_{ak}\tilde{\nu_{k}}P_{k}\frac{L_{a}}{N_{a}}\left(1-\tilde{Q_{kl}}\right)+\\ & & \;\alpha_{kk}\theta \tilde{\nu_{k}}P_{k}\frac{H_{k}}{N_{k}}\left(1-\tilde{Q_{kh}}\right)+\alpha_{ak}\theta \tilde{\nu_{k}}P_{k}\frac{H_{a}}{N_{a}}\left(1-\tilde{Q_{kh}}\right)-\\ & & \;\tau H_{k}-\gamma H_{k}-\mu H_{k},\\ \frac{dR_{kl}}{dt} & = & \gamma L_{k}-\tau R_{kl}-\rho R_{kl}-\mu R_{kl},\\ \frac{dR_{kh}}{dt} & = & \gamma H_{k}-\tau R_{kh}-\rho R_{kh}-\mu R_{kh},\\ \frac{dP_{k}}{dt} & = & \rho R_{kl}+\rho R_{kh}-\\ & & \;\;\alpha_{kk}\tilde{\nu_{k}}P_{k}\frac{L_{k}}{N_{k}}+\alpha_{ak}\tilde{\nu_{k}}P_{k}\frac{L_{a}}{N_{a}}+\alpha_{kk}\theta \tilde{\nu_{k}}P_{k}\frac{H_{k}}{N_{k}}+\alpha_{ak}\theta \tilde{\nu_{k}}P_{k}\frac{H_{a}}{N_{a}}-\tau P_{k}-\tilde{\rho }P_{k}-\mu \;P_{k}. \end{array}$$

We examine how immune duration, susceptibility, infectiousness, and probabilities of developing low-shedding infection alter the number of high- and low-shedding cases. Using data from the CDC [56], we calibrate contact rates so that the model’s total new daily cases match the reported number of probable new cases each day in the United States from 22 January 2020 (day 0) until 16 March 2021 (day 419). We assume that $\alpha_{ak}=\alpha_{aa}=\alpha_{kk}=\alpha$, or that they are in fixed proportions relative to α. At each time step we adjust α so the model matches the daily calibration target. High-shedding and low-shedding cases are produced in children or adults in proportions which are determined by the other parameters in the model (see Table 1 and Table 2). We assume vaccination commences at a constant rate, starting on 16 January 2020 (day 360) and ending on day 460. After 16 March 2021 (day 419), we set α to be constant in time consistent with $R_{0}=1.3$ until vaccination ends, and thereafter contact rates rise to levels consistent with 2.5. We quantify the model’s response by considering (i) cumulative cases of each type by time *t* (ii) active cases of each type at time *t*, (iii) the fraction of infections that are high-shedding at time *t*, and (iv) the fraction of infections up to time *t* that have been high-shedding.

## 3. Results

**Figure 4:**
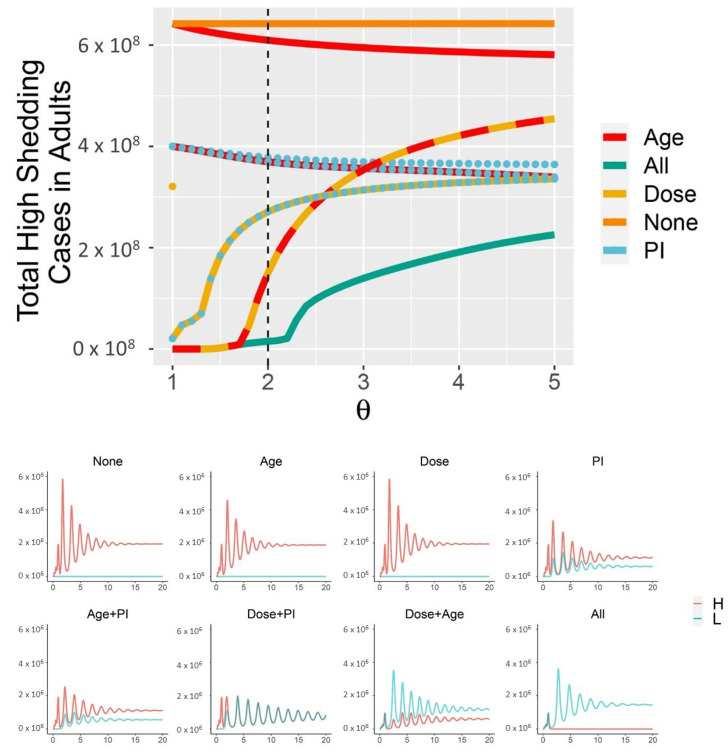
Dose response, partial immunity, and protective effects of youth combine to limit the cumulative number of high-shedding infections which occur within the first 30 years (**top panel**). “Dose” in isolation is identical to “None” for θ>1. Each combination of the three mechanisms produces different dynamics, as shown for θ=2 (**bottom panel**, which corresponds to dotted black line in the **top panel**). High-shedding cases are driven close to extinction only if all three mechanisms are in place (“All mechanisms” in (**bottom panel**)). Cases are shown within adults only. Across all simulations, $\nu_{k}=1/2$, $\Omega =3\times 10^{6}$/day. In the “All” curves, $Q_{ab}=0$, and the other $Q_{xy},\tilde{Q_{xy}}=1$. In the “None” curves, $Q_{xy}=\tilde{Q_{xy}}=0$. Curves with the “Age” mechanism have $Q_{ky}=\tilde{Q_{ky}}=1$. Curves with the “Dose” mechanism have $Q_{xl}=\tilde{Q_{xl}}=1$, and “Dose only” additionally has $Q_{xh}=\tilde{Q_{xh}}=0$ (partial immunity and Age override this latter constraint). Curves with the “PI” mechanism have $\tilde{Q_{xy}}=1$.

The combined effects of partial immunity, age-structure, and dose response effectively control the total number of high-shedding cases across a broad range of infectiousness for the high-shedding phenotype (Figure 4). Subsets of these mechanisms fail to limit high-shedding cases when they are only slightly more infectious than low-shedding cases (Figure 4). Some subsets of mechanisms are more effective than others. In particular, partial immunity and protective youth effects are ineffective to control high-shedding cases without a dose response present, whereas a dose-response combined with a protective youth effect but no partial immunity is at the higher end of effectiveness. Partial immunity is the most effective mechanism in isolation, particularly for large θ (Figure 4). The dose response is the least effective mechanism in isolation. Even with all three mechanisms in place it is impossible to eradicate high-shedding infections if they are too infectious (θ→∞ in Figure 4).

**Figure 5:**
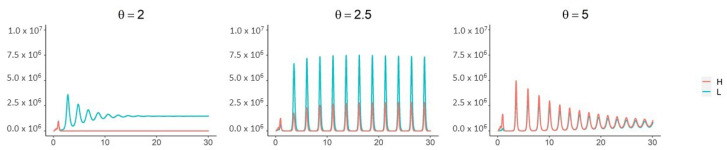
With all three mechanisms in place and children less susceptible to infection ($\nu_{k}=1/2$), a Hopf bifurcation occurs as θ passes through ∼2.3. For θ near 2.5, high-shedding cases return in greater numbers on a biennial cycle and eventually exceed their initial outbreak levels. The magnitude of outbreaks diminishes as θ increases past 2.5 but high-shedding infections make up a greater share of total infections in adults. Periodic solutions do not exist if children are more susceptible than adults ($\nu_{k}>1$). Vertical axes are the number of active infections in adults. Except for θ, all parameters as in Figure 4.

**Figure 6:**
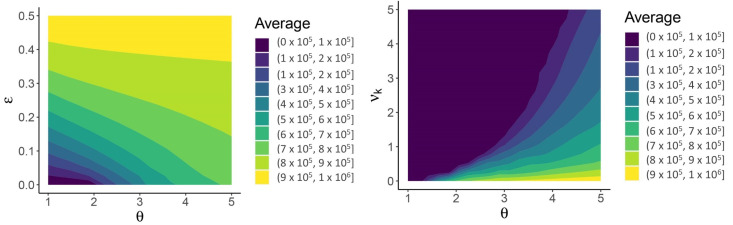
The Sweet Spot for JASC: the three mechanisms in combination limit the long-term average number of high-shedding cases if they generate low-shedding cases with probability close to 100% and provided that high-shedding cases are not too infectious (ϵ≈0,θ≈1, (**left panel**)). Children help mitigate disease severity if they develop low-shedding infections and are more susceptible than adults ($\nu_{k}>1$, (**right panel**)). High-shedding infections can make up half of the total infection prevalence in adults if children do not acquire infection ($\nu_{k}<1$, right panel, and also refer to the θ=5 case in Figure 5). High-shedding infections in adults are rare and periodic outbreaks will not occur if children are equally susceptible ($\nu_{k}=1$). Low-shedding infections in adults further decline if children are more susceptible ($\nu_{k}\to \infty$). Parametrization in the left panel corresponds to $\nu_{k}=1/2$ and the parametrization in the right panel corresponds to ϵ=0. See text for description of the other parameters.

**Figure A3:**
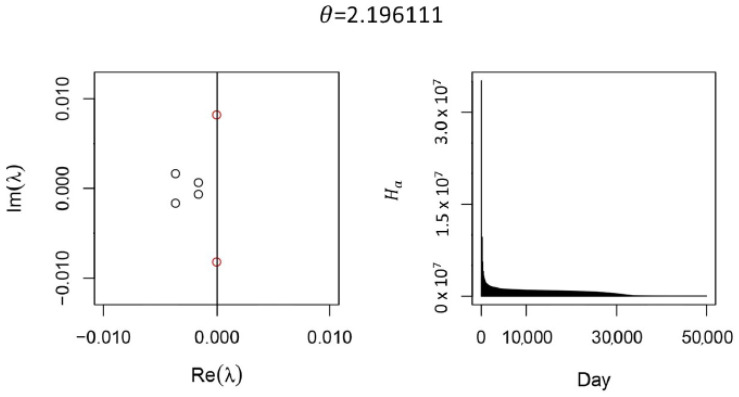
Supercritical Hopf Bifurcation in the Full Model. The red complex eigenpair is about to cross the imaginary axis as θ passes near 2.2, resulting in a supercritical Hopf bifurcation (left). Two real eigenvalues near −0.1 are not shown. Near the bifurcation, transients decay slowly (right). These results were generated from the full model without calibrating to data, without vaccination, $\nu_{k}=1/2$, $Q_{ah}=0$, $Q_{al}=Q_{kl}=Q_{kh}=\tilde{Q_{ah}}=\tilde{Q_{al}}=\tilde{Q_{kl}}=\tilde{Q_{kh}}=1$, and contact rates $\alpha_{aa}=\alpha_{ak}=\alpha_{kk}=\alpha$ were chosen so $R_{0}=2.5$. For simplicity, we have set τ=μ=0. Hopf bifurcations are only present close the limiting case $Q_{ah}=0$, $Q_{al}=Q_{kl}=Q_{kh}=\tilde{Q_{ah}}=\tilde{Q_{al}}=\tilde{Q_{kl}}=\tilde{Q_{kh}}=1$.

**Figure A4:**
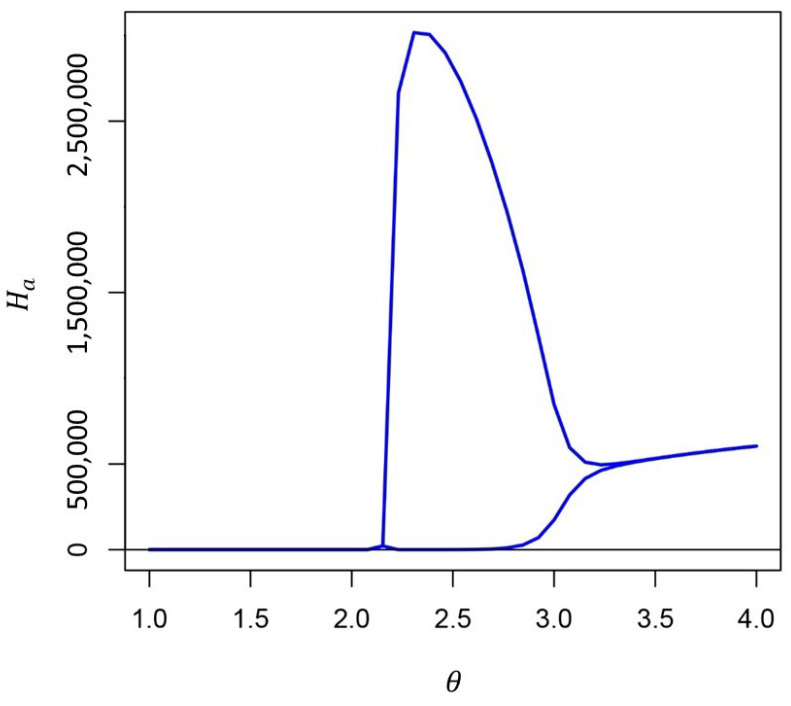
Amplitude of solutions in the Full Model at 10,000 days. The supercritical Hopf bifurcation appears near θ=2.2. For large enough θ the periodic solutions disappear through another supercritical Hopf bifurcation (eigenvalue analysis not shown). These results were obtained from the same parametrization as Figure A3.

When children are less susceptible to infection ($\nu_{k}=1/2$) and all three mechanisms are in place ($Q_{ah}=0$, $Q_{al}=Q_{kl}=Q_{kh}=\tilde{Q_{ah}}=\tilde{Q_{al}}=\tilde{Q_{kh}}=\tilde{Q_{kl}}=0$), a supercritical Hopf bifurcation occurs as θ passes through 2.3 (Figure 5, Figure A3 and Figure A4). This coincides with the corner seen in the “All mechansisms” curve in the top panel of Figure 4. Periodic outbreaks of high-shedding cases in adults occur on a biennial cycle. For θ≈2.5, high-shedding cases decline after the initial outbreak but then return in greater numbers with each cycle until they settle on a long-term amplitude which exceeds that of the initial outbreak (Figure 5). The magnitude of periodic outbreaks diminishes as high-shedding infections become increasingly infectious (θ>2.5) but high-shedding cases persist (Figure 5 and Figure 6). For large enough values of infectiousness θ, the periodic orbits disappear through another supercritical Hopf bifurcation (Figure A4).

Weakening the three mechanisms pushes the system away from JASC. In Figure 6, the parameter ϵ enters into the parametrization as $Q_{ah}=\epsilon ,$$Q_{al}=Q_{kl}=Q_{kh}=\tilde{Q_{ah}}=\tilde{Q_{al}}=\tilde{Q_{kh}}=\tilde{Q_{kl}}=1-\epsilon$, so ϵ>0 constitutes a deviation away from the boundary of 100% efficacy in the three mechanisms. The long-term average number of high-shedding cases in adults decreases as ϵ→0 and θ→1 (Figure 6). Deviating from the boundary of 100% efficacy (ϵ>0) eliminates the Hopf bifurcation, and periodic outbreaks do not occur (not shown).

**Figure 7:**
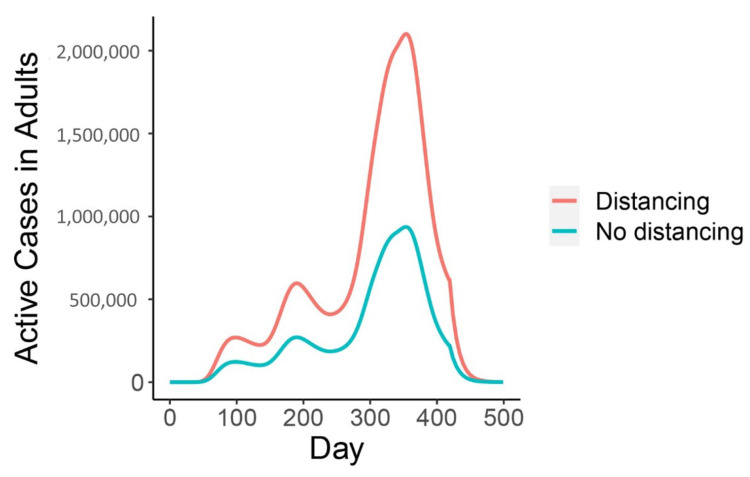
Social distancing which disproportionately reduces contact between children and adults from day 20 to 460 could increase the number of high-shedding cases in adults if children always develop low-shedding infections. The effect is enhanced for a larger vaccination rate. The vaccination period begins on day 360 and ends on 460 (rate of 3 million doses per day). For the “Distancing” curve, $\alpha_{ak}=0$ from days 20–460. For the “No distancing” curve, $\alpha_{ak}$, $\alpha_{aa}$ and $\alpha_{kk}$ are set equal and are calibrated to the incidence data up to day 418, are set to values consistent with $R_{0}=1.3$ from days 418-460, and $R_{0}=2.5$ thereafter. Other parameters: $Q_{ab}=0$, $Q_{am}=Q_{kb}=Q_{km}=\tilde{Q_{ab}}=\tilde{Q_{am}}=\tilde{Q_{kb}}=\tilde{Q_{km}}=1$, and $\nu_{k}=1/2$.

Children help control the number of high-shedding cases in adults as long as there is a dose response (Figure 4 and Figure 6). If they are more susceptible to infection they keep the long-term number of active high-shedding cases low (Figure 6). If children are more susceptible to infection, periodic outbreaks of high-shedding cases in adults do not occur and they can be driven to extinction, or nearly so (not shown). Social distancing that disproportionately reduces contact between children and adults can offset these beneficial effects and potentially increase the number of high-shedding infections in adults (Figure 7).

**Figure 8:**
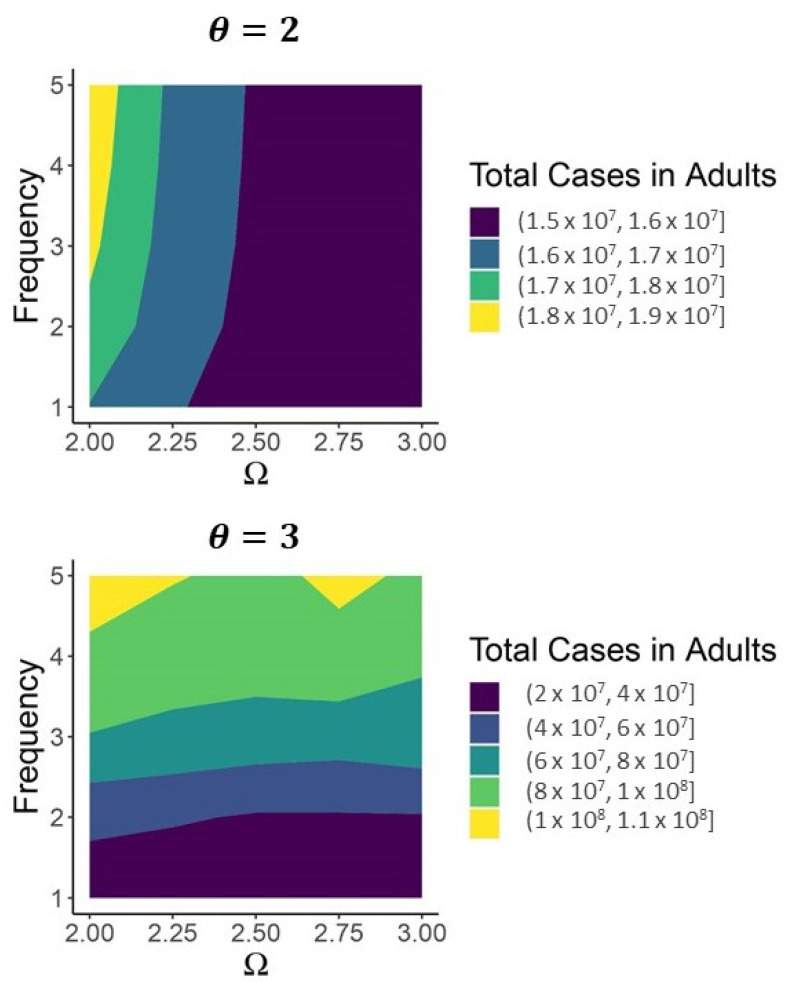
As long as high-shedding cases are not too infectious (θ=2) prolonged vaccination over the next thirty years is not necessary for SARS-CoV-2 to become JASC. If high-shedding cases are sufficiently infectious to cause periodic outbreaks (θ=3) then regular vaccination is necessary to limit high-shedding cases. Vaccination rate Ω is the number of doses (in the millions) administered to susceptible individuals per day during the intense vaccination program which begins on day 360 and lasts until day 460. After day 460, 60% of the population continues to get vaccinated at the corresponding Frequency (in years) along the vertical axis. Contact levels consistent with $R_{0}=1.3$ maintained from day 418 (16 March 2021) up to day 460, after which contact returns to levels consistent with $R_{0}=2.5$. In both panels $Q_{ab}=0$, $Q_{am}=Q_{kb}=Q_{km}=\tilde{Q_{ab}}=\tilde{Q_{am}}=\tilde{Q_{kb}}=\tilde{Q_{km}}=1$, and $\nu_{k}=1/2$.

A sufficiently intense short-term vaccination program obviates the need for subsequent vaccination if high-shedding cases are not too infectious (large Ω in the θ=2 panel in Figure 8). If they are too infectious to disappear from the population then subsequent vaccination can greatly reduce the number of high-shedding cases which occur over a thirty year time period (θ=3 in Figure 8).

## 4. Discussion

Will SARS-CoV-2 become “Just Another Seasonal Coronavirus?” We developed a mathematical model where infections can be “high-shedding”or “low-shedding”, with the latter tending to coincide with mild forms of illness, similar to infections caused by the seasonal coronaviruses. We explore how three mechanisms proposed to alter the severity of COVID-19 might work together to promote avirulence. First, milder cases might produce a smaller number of infectious particles and in turn create milder cases. Second, children have lower susceptibility to severe infection overall and thus provide a source of mild low-shedding infections. Finally, immunity may wane via a partially immune class that, like children, experiences mild low-shedding infections. We implement these in the model by structuring the host population into high and low susceptibility, to represent adults and children, respectively. Each infection phenotype reproduces itself in new susceptible hosts with greater probability, with children and partially immune hosts predisposed for low-shedding infections. Each mechanism, considered in isolation, has the potential to direct the system toward an avirulent regime as long as the high-shedding, severe manifestation of infection is not more infectious than the low-shedding, mild phenotype. However, when these mechanisms act in combination they strongly reduce the number of severe infections.

Because we have evidence for all three mechanisms for SARS-COV-2, it is quite possible that the initial outbreak composed of many severe, high-shedding infections can be followed by an endemic state characterized by mild, low-shedding infections much like the seasonal coronaviruses. Other models predict COVID-19 is likely to persist alongside the seasonal coronaviruses [3], and severity could attenuate over time as the attack rate concentrates in children, who are predisposed for mild illness [65]. Our results show that under the right circumstances, protective effects from age combined with a dose response and partial immunity can accelerate this process. Given the relatively early stage of the pandemic and limited information, it is too early to assess whether this process is indeed under way, and where it will end up. Epidemiological data [66] will provide clues as to whether SARS-CoV-2 is transitioning to JASC according to the mechanisms we have proposed. Data accounting for age, infection severity, vaccination history, and prior infection history (potentially including other viruses) could quantify the strength of the proposed mechanisms. Information on geographic setting or network connectivity will be necessary to place individuals in a context of the degree of immunity and severity to test whether exposure to mild/low-shedding cases is associated with lower disease severity. Further study will also be needed to establish whether individuals with a history of prior infection tend to exhibit milder symptoms when they are reinfected. These analyses will need to account for whether people have received a vaccine, because this could also be associated with less severe illness.

Vaccines add other challenges to this evaluation. They might or might not mimic natural infection in terms of the type and duration of immunity. If disease severity tends to be lower after widespread vaccination, that could indicate that vaccines confer the type of partially protective immunity we considered. The issue is complicated by the multiple vaccine technologies in use (such as mRNA vs viral vector) and by potential interaction with prior infection, that might confer different types of protection for different amounts of time.

Heterogeneous responses to the pandemic across the world provide natural experiments to test whether prior exposure leads to less severe disease. In places where a large fraction of the population experienced natural infection, we might expect future reinfections to tend less severe because prior exposure has conferred some degree of partial immunity. In places which succeeded in keeping infection rates low we might expect future primary infections to tend more severe because the population remains immunologically naive. Of course, widespread vaccination would be expected to alter that outcome.

As with many studies [67], this simple ordinary differential equation model places individuals into a rather small number of categories (adults vs children, partially vs totally immune, low vs high-shedding infection). We do expect our results to be robust to generalization to more finely divided categories, but only further modeling could establish that. This well-mixed model for the U.S. fails to capture spatial dynamics, which could produce entirely different behavior. Similarly, we did not include seasonality in transmission that could alter the speed of transition of SARS-CoV-2 to JASC by clustering cases in time. The models assume constant transition rates between classes, creating exponential sojourn times [54] that are likely not realistic particularly for immunity. Although there is considerable uncertainty about parameter values, we were able to calibrate to existing data and experiment with a wide range of parameter values. Due to limited current knowledge, we ignored heterogeneity in vaccine effects resulting from different technologies, and assumed that vaccine efficacy is homogeneous across the population.

Existing genetic variation in the virus and further genetic mutation may alter the evolutionary path of SARS-CoV-2 in ways that our model cannot predict. In particular, new variants that escape partial immunity could short-circuit the process proposed here. Interactions with the seasonal coronaviruses or other upper respiratory viruses could create unexpected feedbacks [3]. In addition, the immune system could select for more virulent genetic variants within hosts, as seems to be the case with malaria [68]. On a longer timescale than we have considered, the human immune system and SARS-CoV-2 may coevolve in the context of a genetic arms race rendering pathogen more virulent and host more resistant [69].

Emerging pathogens can be quite virulent, but as they transition to members of the larger ecosystem they often tend toward avirulence. The role the immune system plays in this transition is unclear. The COVID-19 pandemic provides us with an opportunity to better understand how it might facilitate the evolution of disease. If it mitigates disease severity in a dose-dependent manner like we have described, then our results suggest that mild or asymptomatic infections by SARS-CoV-2 will become typical. Although viral evolution and interactions with vaccines complicate the picture, we maintain hope that SARS-CoV-2 will become the fifth seasonal coronavirus.

## Figures and Tables

**Table 1:** State Variables for the Full Model.

Variable	Description	Initial Condition
$S_{x}$	Susceptible adults and children	271,833,136 and 56,406,387 [55]
$P_{x}$	Partially resistant adults, children	0
$L_{x}$	Low-shedding infected adults, children	0
$H_{x}$	High-shedding infected adults, children	1, 0
$R_{xy}$	Resistant adults/children	0
	after low-shedding/high-shedding infection	

State variables describe the U.S. population, with children defined to be 14 years of age or younger [20,55,57]. The total population size is 328,239,523 [55]. Subscripts with *x* index age (*a* or *k* for adults or children) and the extra *y* in the subscript for Resistant individuals denotes the type of infection experienced (*h* or *l* for high- or low-shedding). The epidemic is seeded by a single high-shedding adult.

**Table 2:** Parameters for the full model.

Parameter	Description	Value
$\tilde{\rho }$	Loss rate of partial immunity	$\frac{1}{2}$ per year [58,59,60,61]
ρ	Loss rate of sterilizing immunity	$\frac{1}{2}$ per year [58,59,60,61]
$\alpha_{ak}$	Contact rate between adults and children	Calibrated to
		daily incidence data
		or $R_{0}=1.3,2.5$ [56,62]
$\alpha_{aa}$	Contact rate between adults	Calibrated to
		daily incidence data
		or $R_{0}=1.3,2.5$ [56,62]
$\alpha_{kk}$	Contact rate between children	Calibrated to
		daily incidence data
		or $R_{0}=1.3,2.5$ [56,62]
θ	Infectiousness of high-shedding cases
	relative to low-shedding cases	[1, 5]
τ	Maturation rate	$\frac{1}{14}$ per year [55]
μ	Death rate	$\frac{1}{80}$ per year [55]
γ	Recovery rate from infection	$\frac{1}{10}$ per day [63,64]
$\nu_{k}$	Susceptibility, S child	[0, 5]
$\tilde{\nu_{a}}$	Susceptibility, P adult	$\frac{1}{2}$
$\tilde{\nu_{k}}$	Susceptibility, P child	$\frac{1}{2}$ $\nu_{k}$
$Q_{xy}$	Probability an individual in $S_{x}$ develops	[0, 1]
	low-shedding infection $L_{x}$ upon contact with	
	an infection of type *y*	
$\tilde{Q_{xy}}$	Probability an individual in $P_{x}$ develops	[$Q_{xy}$,1]
	low-shedding infection $L_{x}$ upon contact with	
	an infection of type *y*	
Ω	Daily vaccination rate	[$10^{6}$, $3\times 10^{6}$]
	from days 360–460	

**Parameter values used in the full model.** Rates of immune loss are chosen so the duration of immunity to SARS-CoV-2 lies between the duration reported for SARS and the seasonal coronaviruses [58,59,60,61]. The average infectious period is chosen to coincide with estimated durations of infectiousness [63,64]. Contact rates are either (i) calibrated to match daily incidence, or (ii) held at fixed values to coincide with a given $R_{0}$ value, as explained at the end of the Methods section. Susceptibility parameters were chosen so partial immunity reduces the probability of infection by 50% in all of the figures to follow, but main results are not sensitive to the particular value (Figure A1). Other parameters which are not fixed across simulations are unknown and are varied over the ranges indicated by the intervals across simulations. Relationships between these parameters and model outputs are presented within the figures in Results.

**Figure A2:**
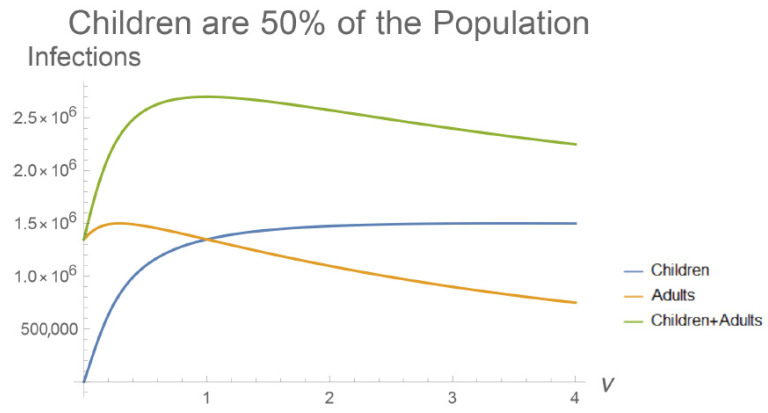
The values of $I_{a}^{*}$ and $I_{k}^{*}$ from the age-structured model plotted against ν, the relative susceptibility of children, who make up half the population. The curves are qualitatively similar to those in Figure 3 but the relationships are more pronounced since children make up a greater share of the population.

## Data Availability

COVID-19 case count data obtained from https://catalog.data.gov/dataset/united-states-covid-19-cases-and-deaths-by-state-over-time (accessed on 18 March 2021). Demographic data for the United States obtained from https://www.census.gov/newsroom/press-kits/2020/population-estimates-detailed.html (accessed on 18 March 2021).

## Abbreviations

The following abbreviations are used in this manuscript:

JASC	Just Another Seasonal Coronavirus

## Appendix A

### Appendix A.1. Full Steady State Solution for the Model with Two Infection Types

The exact solution for the steady-state is

$$\begin{array}{cc} S^{*} & =N\frac{1}{R_{0}}\frac{\tilde{\rho }}{\tilde{\rho }+{\left(\frac{1}{\rho }+\frac{1}{\gamma }\right)}^{-1}\left(R_{0}-1\right)},\\ P^{*} & =N\frac{1}{R_{0}}\frac{{\left(\frac{1}{\rho }+\frac{1}{\gamma }\right)}^{-1}\left(R_{0}-1\right)}{\tilde{\rho }+{\left(\frac{1}{\rho }+\frac{1}{\gamma }\right)}^{-1}\left(R_{0}-1\right)},\\ L^{*} & =N\frac{\rho }{\gamma +\rho }\left(1-\frac{1}{R_{0}}\right)\frac{{\tilde{Q}}_{h}{\left(\frac{1}{\rho }+\frac{1}{\gamma }\right)}^{-1}\left(R_{0}-1\right)+Q_{h}\tilde{\rho }}{\left(1+{\tilde{Q}}_{h}-{\tilde{Q}}_{l}\right){\left(\frac{1}{\rho }+\frac{1}{\gamma }\right)}^{-1}\left(R_{0}-1\right)+\left(1+Q_{h}-Q_{l}\right)\tilde{\rho }},\\ H^{*} & =N\frac{\rho }{\gamma +\rho }\left(1-\frac{1}{R_{0}}\right)\frac{\left(1-{\tilde{Q}}_{l}\right){\left(\frac{1}{\rho }+\frac{1}{\gamma }\right)}^{-1}\left(R_{0}-1\right)+\left(1-Q_{l}\right)\tilde{\rho }}{\left(1+{\tilde{Q}}_{h}-{\tilde{Q}}_{l}\right){\left(\frac{1}{\rho }+\frac{1}{\gamma }\right)}^{-1}\left(R_{0}-1\right)+\left(1+Q_{h}-Q_{l}\right)\tilde{\rho }},\\ R^{*} & =N\frac{\gamma }{\gamma +\rho }\left(1-\frac{1}{R_{0}}\right). \end{array}$$

The approximation in the main text is obtained by assuming that $\gamma >>\rho ,\tilde{\rho }$. Adding $H^{*}+L^{*}$ gives the expression for total infection prevalence in the main text. The long-term amount of partial immunity is weighted by the product of $R_{0}-1$ and the total amount of time spent in the infected and removed states, ${\left(\frac{1}{\rho }+\frac{1}{\gamma }\right)}^{-1}$, reflecting the fact that an infection with a relatively small $R_{0}$ will not spread to much of the population.

### Appendix A.2. Simplifying the Full Model

When there are no differences between high- and low-shedding infections, and children and adults are identical, the model simplifies. The following sets of parameters can be equated:

$$\begin{array}{cc} & Q=Q_{ah}=Q_{al}=Q_{kh}=Q_{kl}=\tilde{Q_{ah}}=\tilde{Q_{al}}=\tilde{Q_{kh}}=\tilde{Q_{kl}}\\ & \nu =\tilde{\nu_{a}}=\nu_{a}=\tilde{\nu_{k}}=\nu_{a}\\ & E=E_{al}=E_{ah}=E_{kl}=E_{kh}\\ & \theta =\theta_{l}=\theta_{h}\\ & \alpha =\alpha_{aa}=\alpha_{kk}=\alpha_{ak}\\ & \gamma =\gamma_{al}=\gamma_{ah}=\gamma_{kl}=\gamma_{kh}\\ & I=H_{a}+L_{a}+H_{k}+L_{k}\\ & S=S_{a}+S_{k}+P_{a}+P_{k} \end{array}$$

The system of equtaions simplifies to

$$\begin{array}{c} \frac{dI}{dt}=\alpha \theta \nu S\left(\frac{L_{k}+H_{k}}{N_{k}}+\frac{L_{a}+H_{a}}{N_{a}}\right)-\gamma I \end{array}$$

With age structure, but no difference between high- and low-shedding infection, the parameterization becomes

$$\begin{array}{cc} & Q_{a}=Q_{ah}=Q_{al}=\tilde{Q_{ah}}=\tilde{Q_{al}}\\ & Q_{k}=Q_{kh}=Q_{kl}=\tilde{Q_{kh}}=\tilde{Q_{kl}}\\ & \nu_{a}=\tilde{\nu_{a}}\\ & \nu_{k}=\tilde{\nu_{k}}\\ & E_{a}=E_{al}=E_{ah}\\ & E_{k}=E_{kl}=E_{kh}\\ & \theta =\theta_{l}=\theta_{h}\\ & \gamma_{a}=\gamma_{al}=\gamma_{ah}\\ & \gamma_{k}=\gamma_{kl}=\gamma_{kh} \end{array}$$

The system of equations can be reduced to

$$\begin{array}{ccc} \frac{dL_{a}}{dt}+\frac{dH_{a}}{dt} & = & \theta \nu_{a}\left(S_{a}+P_{a}\right)\left(\alpha_{ak}\frac{L_{k}+H_{k}}{N_{k}}+\alpha_{aa}\frac{L_{a}+H_{a}}{N_{a}}\right)-\gamma_{a}\left(L_{a}+H_{a}\right) \end{array}$$

$$\begin{array}{ccc} \frac{dL_{k}}{dt}+\frac{dH_{k}}{dt} & = & \theta \nu_{k}\left(S_{k}+P_{k}\right)\left(\alpha_{kk}\frac{L_{k}+H_{k}}{N_{k}}+\alpha_{ak}\frac{L_{a}+H_{a}}{N_{a}}\right)-\gamma_{k}\left(L_{k}+H_{k}\right) \end{array}$$

Ignoring age structure but allowing for differences between high- and low-shedding infection, the parameterization can be simplified to

$$\begin{array}{cc} & Q_{l}=Q_{al}=Q_{kl}\\ & Q_{h}=Q_{ah}=Q_{kh}\\ & \tilde{Q_{l}}=\tilde{Q_{al}}=\tilde{Q_{kl}}\\ & \tilde{Q_{h}}=\tilde{Q_{ah}}=\tilde{Q_{kh}}\\ & \alpha =\alpha_{aa}=\alpha_{ak}=\alpha_{kk}\\ & \tilde{\nu }=\tilde{\nu_{a}}=\tilde{\nu_{k}}\\ & \nu =\nu_{a}=\nu_{k}\\ & E_{l}=E_{al}=E_{kl}\\ & E_{h}=E_{ah}=E_{kh}. \end{array}$$

The system of differential equations simplifies to

$$\begin{array}{ccc} \frac{dL_{a}}{dt}+\frac{dL_{k}}{dt} & = & \alpha [\nu \left(S_{a}+S_{k}\right)\left(\theta_{l}Q_{l}\left(\frac{L_{k}}{N_{k}}+\frac{L_{a}}{N_{a}}\right)+\theta_{h}Q_{h}\left(\frac{H_{k}}{N_{k}}+\frac{H_{a}}{N_{a}}\right)\right)+\\ & & \;\tilde{\nu }\left(P_{a}+P_{k}\right)(\theta_{l}\tilde{Q_{l}}\left(\frac{L_{k}}{N_{k}}+\frac{L_{a}}{N_{a}}\right)+\theta_{h}\tilde{Q_{h}}\left(\frac{H_{k}}{N_{k}}+\frac{H_{a}}{N_{a}}\right)]\\ & & \;-\gamma_{l}\left(L_{a}+L_{k}\right) \end{array}$$

$$\begin{array}{ccc} \frac{dH_{a}}{dt}+\frac{dH_{k}}{dt} & = & \alpha [\nu \left(S_{a}+S_{k}\right)\left(\theta_{l}\left(1-Q_{l}\right)\left(\frac{L_{k}}{N_{k}}+\frac{L_{a}}{N_{a}}\right)+\theta_{h}\left(1-Q_{h}\right)\left(\frac{H_{k}}{N_{k}}+\frac{H_{a}}{N_{a}}\right)\right)+\\ & & \;\tilde{\nu }\left(P_{a}+P_{k}\right)\left(\theta_{l}\left(1-\tilde{Q_{l}}\right)\left(\frac{L_{k}}{N_{k}}+\frac{L_{a}}{N_{a}}\right)+\theta_{h}\left(1-\tilde{Q_{h}}\right)\left(\frac{H_{k}}{N_{k}}+\frac{H_{a}}{N_{a}}\right)\right)]\\ & & \;-\gamma_{h}\left(H_{a}+H_{k}\right). \end{array}$$
